# Neoadjuvant Cetuximab Leading to a Complete Pathologic Response in Locally Advanced Lip Squamous Cell Carcinoma

**DOI:** 10.7759/cureus.75000

**Published:** 2024-12-02

**Authors:** Ruba Alchaikh Hassan, Zahra Gafarzadeh, Shiva Salmasi, David M Hyams, Constantin A Dasanu

**Affiliations:** 1 Internal Medicine, Eisenhower Health, Rancho Mirage, USA; 2 Internal Medicine, Eisenhower Medical Center, Rancho Mirage, USA; 3 Surgical Oncology, Eisenhower Medical Center, Rancho Mirage, USA; 4 Oncology and Hematology, Lucy Curci Cancer Center, Eisenhower Health, Rancho Mirage, USA

**Keywords:** cemiplimab, cetuximab, head and neck cancer, lip squamous cell carcinoma, neoadjuvant

## Abstract

Vermillion lip squamous cell carcinoma (SCC) is a rare cancer, currently grouped together with the cutaneous lip under the cutaneous squamous cell carcinoma (cSCC) system. Herein, we present a case of an 81-year-old male with locally advanced lower lip SCC involving the vermillion who achieved a complete pathologic response to neoadjuvant cetuximab after the failure of the programed cell death protein-1 (PD-1) inhibitor, cemiplimab. He was followed with clinical observation, with special attention to skin/mucosal surfaces. At the 24-week follow-up visit, he had no signs of recurrence and was very content with the surgical and cosmetic outcome. To our knowledge, there have been no documented cases of successful use of cetuximab as a neoadjuvant treatment for cSCC of the vermilion lip to date. This case highlights the potential role of cetuximab in this setting, where we believe clinical trials are warranted.

## Introduction

Treatment of lip cancer poses significant challenges due to its unique anatomical location, which can result in both cosmetic and functional impairments. Standard treatments for early-stage lip cancer typically involve surgery or radiotherapy, while more advanced lesions require a multidisciplinary approach and a combination of surgery, radiotherapy, and/or systemic therapy [1]. Neoadjuvant approach represents a viable alternative when these conventional modalities, if used upfront, might yield suboptimal outcomes [2]. Cemiplimab has generally shown favorable outcomes in the neoadjuvant setting for advanced cutaneous squamous cell carcinoma (cSCC) [2]. Cetuximab is a human-murine chimeric monoclonal IgG1 antibody approved for the treatment of head and neck squamous cell carcinoma (HNSCC) [2]. This epidermal growth factor receptor (EGFR) inhibitor showed encouraging results when combined with systemic chemotherapy for recurrent, metastatic or unresectable oropharyngeal cancer [3]. However, there is very limited data regarding its clinical benefit in the neoadjuvant setting. Herein, we present a patient with locally advanced lip SCC successfully treated with cetuximab in the neoadjuvant therapy.

## Case presentation

**Figure 1:**
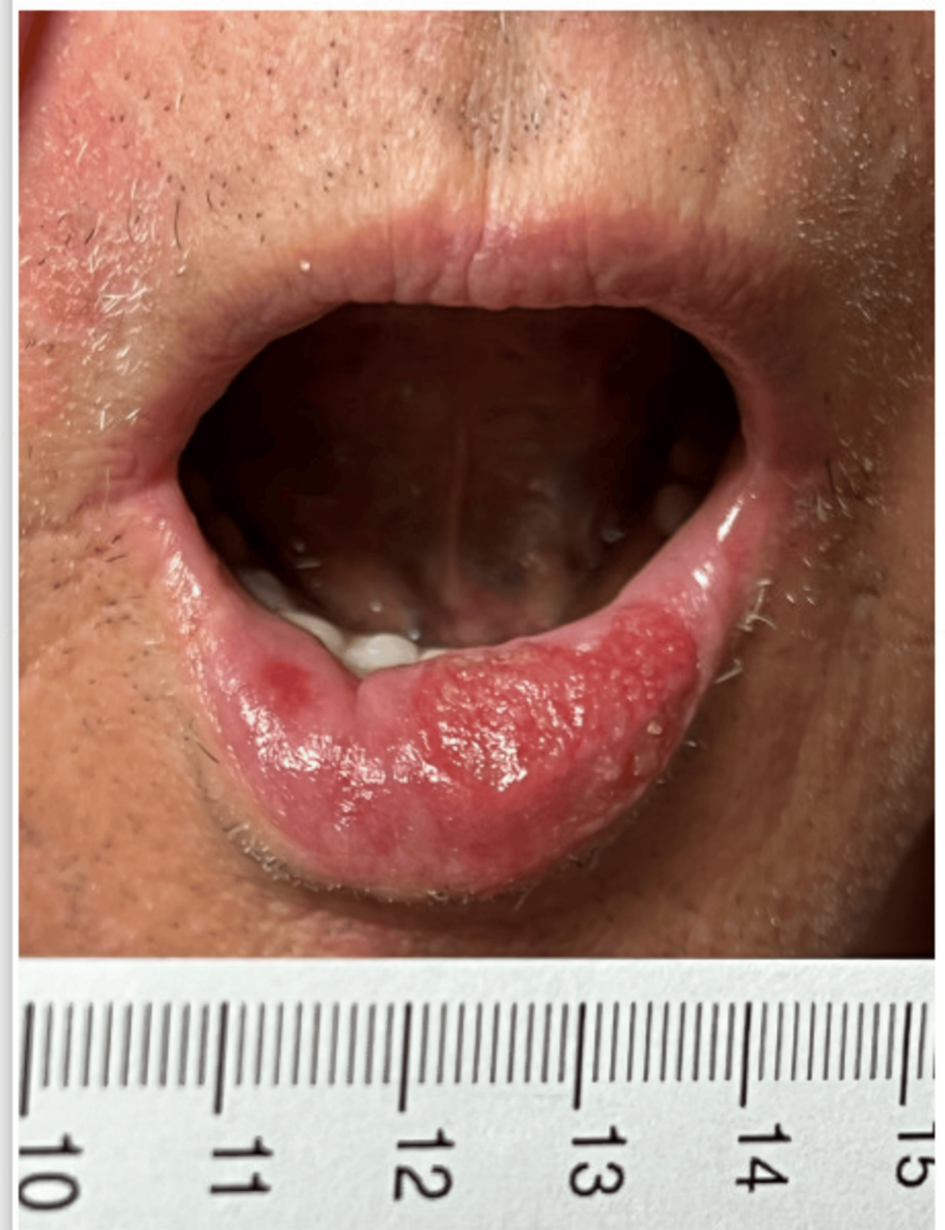
Anterior view of the lower lip at the initial visit showing an erythematous ulcerated mass on the vermilion measuring 2.6 x 1.4 x 0.8 cm in the index patient.

**Figure 2:**
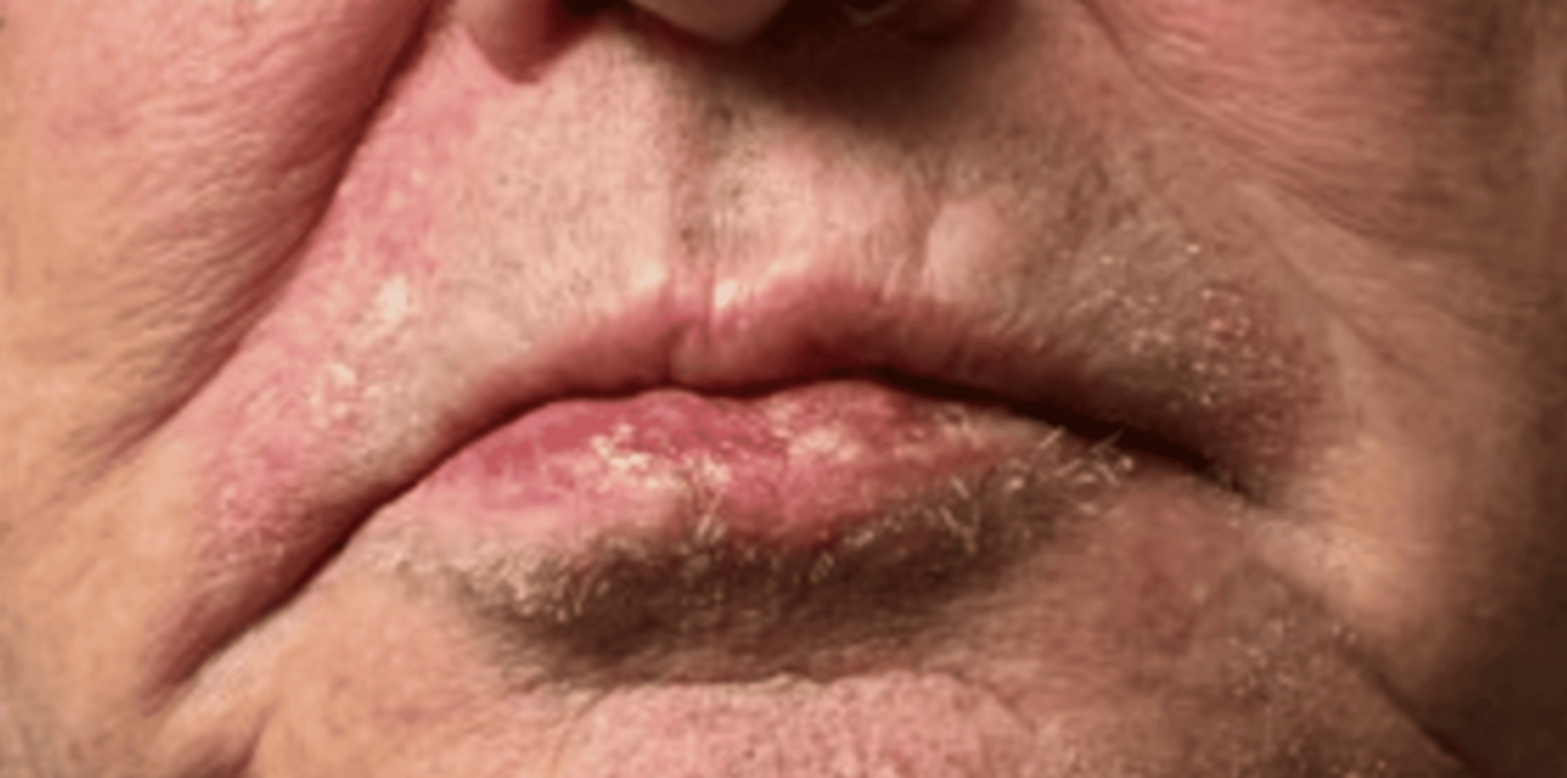
Anterior view of the lower lip after neoadjuvant cetuximab showing a significant reduction in tumor size to 0.6 x 0.5 x 0.5 cm surrounded by a 1.5 cm area of an inflammatory reaction.

**Figure 3:**
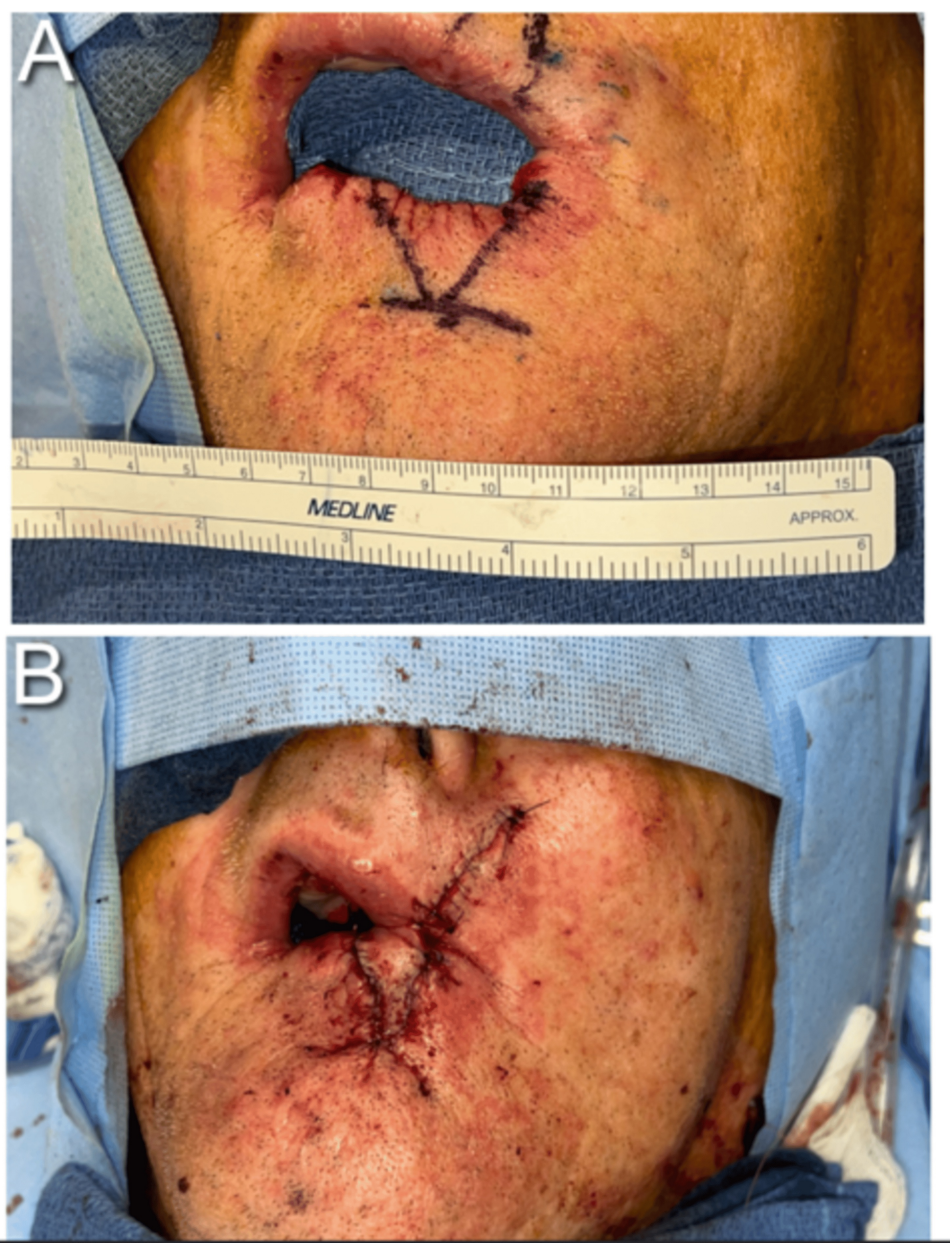
(A, B) Intraoperative views showing a surgical incision of approximately 2 cm in length, performed to achieve a wide excision of the 0.6 x 0.5 x 0.5 cm lesion. The excision was followed by immediate lower lip reconstruction in the index patient.

**Figure 4:**
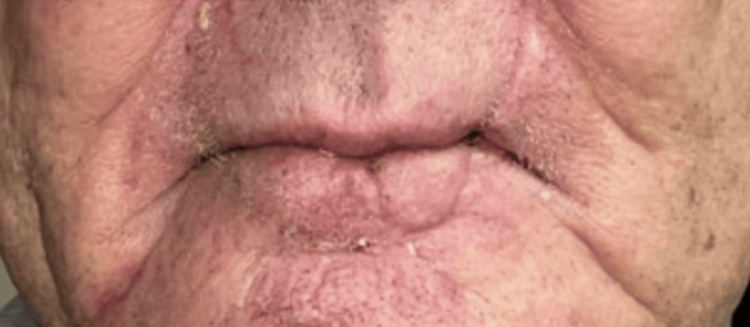
Anterior view of the lower lip at 24-week follow-up visit showing a postoperative scar, with no signs of disease recurrence.

An 81-year-old Caucasian man was referred by a surgical oncologist in January 2024 regarding neoadjuvant therapy after he was diagnosed with unresectable SCC of the lower lip. The patient initially noticed a small, asymptomatic lump on his lower lip in 2022, which progressively developed into a tender, firm vermillion plaque measuring 2.6 cm, with ulceration and intermittent bleeding. This lesion extended asymmetrically towards the left corner of the mouth (Figure 1). Physical examination showed no evidence of intraoral lesions or regional lymphadenopathy. A contrast-enhanced computed tomography (CT) scan of the head and neck showed a 2.8 x 1.5 x 0.9 cm lesion involving lower lip with irregular margins, without evidence of enlarged regional nodes. Shave biopsy showed moderately differentiated SCC, with tumor involvement extending to both deep and lateral margins. According to the tumor-node-metastasis (TNM) staging system, the lesion was classified as cT2 cN0 Mx [4]. Past medical history was significant for coronary artery disease, asthma, hypertension, dyslipidemia, benign prostatic hyperplasia, and gastroesophageal reflux disease. His medications included aspirin, rosuvastatin, ezetimibe, metoprolol, amlodipine, esomeprazole, and an albuterol inhaler PRN. The patient, a former mechanic, had a 30-pack-year smoking history but quit smoking at the age of 60. He denied alcohol excess, injuries to the lip, or exposure to radiation. Family history was unremarkable. Our specialty and subspecialty teams thoroughly reviewed the patient's case, including medical oncology, surgical oncology, oral surgery and radiation oncology. In addition, the case was discussed extensively at the local multidisciplinary tumor board. Upfront radiation therapy or surgical intervention were not favored given the relatively large size of the lesion, and the potential for significant cosmetic and functional impairments. Neoadjuvant therapies including chemotherapy and programmed cell death protein-1 (PD-1) inhibitors were discussed. Using the Chemotherapy Risk Assessment Scale for High-Age Patients (CRASH) Score, the patient was found to be at intermediate-high risk with a score of 8 points on the CRASH scale [5]. The decision was made to proceed with neoadjuvant PD-1 inhibitor cemiplimab given the patient’s multiple comorbidities, potential toxicity of chemotherapy and his personal preference. The patient received intravenous (IV) cemiplimab 350 mg once every three weeks, for a total of three cycles from February to April 2024; however, clinical evaluation showed progression of the lesion. A subsequent positron emission tomography/computed tomography (PET/CT) scan in April 2024 showed standardized uptake value (SUV) of 6.4 in the lower lip with no evidence of regional metastases. Subsequently, the patient received IV cetuximab 400 mg loading dose, followed by two more weekly doses at 250 mg from April to May 2024. During treatment, he developed a painful, grade 2 maculopapular rash on his upper chest and back, along with a tingling sensation in the lips. These symptoms were effectively managed with topical colloidal oatmeal solution twice daily and viscous lidocaine 2% as needed. A clinical evaluation after the third dose of cetuximab indicated a significant decrease in the size of the lesion to 0.6 x 0.5 x 0.5 cm (Figure 2). The patient subsequently underwent radical tumor resection in May 2024. Immediate lip reconstruction utilizing a mucocutaneous cross-lip flap to transfer tissue from the upper lip to the lower lip and chin was performed (Figures 3A, 3B). Histological examination revealed no residual disease, and the sentinel lymph node biopsy was negative. At a follow-up visit in June 2024, the patient had no surgical complications. At a 24-week follow-up, he had no signs of recurrence and was very satisfied with the surgical outcome and physical appearance (Figure 4).

## Discussion

Lip SCC is the most common malignancy affecting the lip and accounts for up to 12% of all malignancies in the head and neck region and up to 25% of oral cancers [6]. Lip SCC has a predilection for the lower lip as opposed to the upper lip [6]. The lip is composed of mucosal membrane, vermilion, and cutaneous surface which makes it vulnerable to two different carcinomas: cSCC and oral-mucosal SCC [7]. According to The American Joint Committee on Cancer Staging Manual (AJCC) seventh edition, SCC on the cutaneous surface of the lip was categorized under the cSCC system, whereas SCC on the vermilion lip was classified within the lip and oral cavity system [8]. In AJCC eighth edition, the SCC on the vermilion lip was reclassified and now grouped with the cutaneous lip under the cSCC system [4,8]. Risk factors for lip SCC include male gender; older age; fair complexion; exposure to ultraviolet light; tobacco smoking; excessive alcohol consumption; infection with human papillomavirus (HPV) and immunosuppression [1]. Lip SCC generally has a favorable prognosis due to early detectability and treatment. Nevertheless, the prognosis may significantly worsen in the setting of large lesions, recurrence, treatment failure, or the presence of metastases [9]. The current treatment modalities for lip SCC include surgery, radiotherapy, and systemic therapy, which may be administered individually or in combination based on the tumor stage and patient comorbidities [9]. Early-stage cancers are typically managed with single-modality therapy such as surgery or radiation therapy, while more advanced-stage cancers may necessitate combination therapy including surgery followed by radiation therapy and/or systemic therapy [9]. By definition, a neoadjuvant approach aims to reduce the size of the tumor to make it suitable for other potentially curative treatments. This approach is most commonly used in cases where there is a significant risk of functional and cosmetic defects from immediate surgery or radiotherapy due to the tumor's size or location [2]. The promising outcomes linked to the use of immune checkpoint inhibitors (ICIs) for advanced cSCC have created significant interest in their potential application as neoadjuvant therapy [10]. In a single-institution pilot study of 20 patients with cSCC, 70% of patients achieved either a complete or a partial pathologic response after receiving two cycles of cemiplimab [11]. Similar pathologic response rates were observed in a multicenter, international phase 2 trial involving 79 patients with resectable stage II-IV cSCC, where 51% of patients achieved a complete response and 13% a partial response after receiving four cycles of cemiplimab [12]. Notably, in both studies a majority of patients had cSCC located in the head and neck region [11,12]. These findings suggest clinical benefits with cemiplimab in the neoadjuvant setting for advanced-stage, resectable cSCC. However, in non-responders to cemiplimab such as our patient, there are currently no standard guidelines to direct the management. The role of EGFR signaling in epithelium-derived neoplasms has been extensively researched, leading to the development of several EGFR inhibitors. Cetuximab, a chimeric IgG1-subclass monoclonal antibody, binds to the extracellular domain of EGFR, which prevents the activation of its intracellular domain and the subsequent tyrosine kinase-dependent signal transduction pathway [3]. In addition, as an IgG1 molecule, it also stimulates antibody-dependent cell cytotoxicity [3]. In 2006, a randomized phase III study conducted by Bonner et al. [13] showed that adding cetuximab to radiotherapy improved locoregional control and overall survival in more than 400 patients with locally advanced cancers of oropharynx, hypopharynx, and larynx (HNSCC). As a result, the US FDA approved cetuximab in this setting in combination with radiotherapy as a valid alternative to standard chemoradiotherapy (CRT). However, its use has been limited to patients considered unfit for CRT due to its inferiority as suggested by meta-analyses and subsequent clinical trials [3]. Furthermore, two-phase III studies evaluating cetuximab-radiotherapy vs CRT in HPV-positive locally advanced oropharyngeal SCC (De-ESCALaTE and RTOG 1016) showed inferior overall survival with the former, and therefore, CRT remains the standard of care in this setting [14,15]. For patients who are not ideal candidates for chemotherapy due to factors such as advanced age, comorbidities, or the potential for significant side effects, immunotherapy and targeted therapy offer valuable alternative treatment options. This is particularly pertinent for frail patients or those who demonstrate high scores on predictive tools for chemotherapy toxicity, such as the CRASH score, as seen with our patient. In addition, the patient preference for the type of therapy should always be taken into consideration. In the neoadjuvant context, Reigneau et al. [16] investigated cetuximab use in 34 patients with unresectable cSCC either in combination with cisplatin or carboplatin and 5-fluorouracil (5-FU) or as monotherapy when platinum with 5-FU therapy was contraindicated. After nine weeks, 92% of the patients who received cetuximab plus chemotherapy and 55% of the patients who received only cetuximab became eligible for surgery [16]. The median age of nine patients who were only treated with cetuximab was 86 and among these patients, three patients were staged by TNM as stage II and the other six patients had stage III disease [16]. Although our patient’s outcome aligns with these results in showing the clinical benefits of using neoadjuvant cetuximab in cSCC, several key differences distinguish our report. Firstly, all the patients in the Reigneau et al. [16] study had highly keratinized skin lesions in areas including the cheek, nose, forehead, eyelid, vertex, and temple, while our patient presented with vermilion lip involvement which carried a greater risk of nodal metastases when compared with cSCCs arising in more keratinized regions with thicker subcutaneous fat layer [8]. Another important distinction is that our patient had previously been treated with a PD-1 inhibitor, which raises the question of whether cetuximab's efficacy might be enhanced when used sequentially with PD-1 inhibitors. Cetuximab generally has a favorable safety profile, even in context of advanced age and multiple comorbidities. Skin reactions develop in up to 80% of patients and present as an acne-like lesions [17]. This rash tends to develop within the first few weeks of therapy and resolve over time without sequelae. The positive outcome in our patient is also consistent with the suggested link between cetuximab-related skin reaction and enhanced treatment efficacy [17].

## Conclusions

We present a successful outcome in a patient treated with neoadjuvant cetuximab therapy for vermilion lip cSCC. Using this approach, he had a complete response to cetuximab. This case highlights the need for further cetuximab evaluation in the neoadjuvant setting in clinical trials. In addition, further investigation regarding the sequential use of PD-1 inhibitors and cetuximab is needed to clarify the potential synergism between these agents.
